# Review of Perdita subgenus Procockerellia Timberlake (Hymenoptera, Andrenidae) and the first *Perdita* gynandromorph

**DOI:** 10.3897/zookeys.712.14736

**Published:** 2017-10-31

**Authors:** Zachary M. Portman, Terry Griswold

**Affiliations:** 1 Utah State University, 5305 Old Main Hill, Logan, UT 84322, USA; 2 USDA ARS Pollinating Insects Research Unit, 5310 Old Main Hill, Logan UT 84322, USA

**Keywords:** Apoidea, *Allomacrotera*, *Stephanomeria*, scopal hairs, distribution

## Abstract

A systematic study of Perdita subgenus Procockerellia Timberlake and the related subgenus Allomacrotera Timberlake results in the synonymy of the latter with the former, and two specific synonymies: Perdita (Hexaperdita) glamis Timberlake is a junior synonym of Perdita (Procockerellia) stephanomeriae Timberlake, while Perdita (Procockerellia) brachyglossa Timberlake is a junior synonym of Perdita (Cockerellia) imbellis Timberlake. Perdita (Procockerellia) moldenkei Timberlake is moved to subgenus Cockerellia Ashmead. A revised subgeneric diagnosis and key to the three included species are provided. Diagnoses of species are updated with novel characters; distributions and biological data are expanded. A gynandromorph of P. (Procockerellia) moabensis Timberlake, the first known in the genus *Perdita*, is reported.

## Introduction

The panurgine genus *Perdita* Smith, 1853 (Hymenoptera: Andrenidae: Panurginae) is the most speciose bee genus in North America with 636 currently recognized species and 127 additional subspecies (Portman et al. 2016b). It is also diverse at the subgeneric level with 17 subgenera currently recognized (Michener 2007). Among these, the subgenera *Procockerellia* Timberlake, 1954 and *Allomacrotera* Timberlake, 1960 have had a complicated and intertwined taxonomic history.

*Procockerellia* was originally described by Timberlake (1954) to include two species: Perdita (Procockerellia) albonotata Timberlake, 1954 (type species) and P. (P.) stephanomeriae Timberlake, 1954. Perdita (P.) excellens Timberlake, 1958 was subsequently described (Timberlake 1958). Upon the discovery of the male of P. (P.) stephanomeriae, Timberlake (1960) split *Procockerellia*, moving P. (P.) stephanomeriae into the new subgenus Allomacrotera. Later, Timberlake (1971) described P. (P.) brachyglossa Timberlake, 1971 and P. (P.) moabensis Timberlake, 1971. Then, Timberlake (1980) described P. (P.) moldenkei Timberlake, 1980 and moved P. (P.) moabensis into *Allomacrotera* due to the discovery of the male. More recently, P. (P.) excellens was synonymized with P. (Xeromacrotera) cephalotes (Cresson, 1878) by Portman et al. (2016a).

To summarize the taxonomic status, three species currently constitute *Procockerellia*: P. (P.) albonotata, P. (P.) brachyglossa, and P. (P.) moldenkei, while two species constitute *Allomacrotera*: P. (A.) moabensis and P. (A.) stephanomeriae.

Here, we assess the taxonomic status of these subgenera and revise the included species. Synonymies and changes presented here result in a single subgenus, *Procockerellia*, containing three species: P. (P.) albonotata, P. (A.) moabensis, and P. (A.) stephanomeriae. These changes reduce the total number of *Perdita* subgenera to 16 and the number of species to 634. A revised key to species of *Procockerellia*, and updated species accounts are presented. Lastly, during the course of this study, a gynandromorph of *P. moabensis* was discovered; its aberrant morphology is described. This specimen is the first described gynandromorph in the genus *Perdita*.

## Methods

Morphological terms follow Michener (2007). The metasomal terga and sterna are abbreviated to T and S, respectively. Specimens were examined using a Leica MZ12 microscope and images and measurements were taken with Keyence VHX-500 and VHX-5000 Digital Imaging Systems. Scanning electron microscope images were taken with a Quanta FEG 650 Scanning Electron Microscope. Images were compiled into plates using Adobe Photoshop CS5 and maps were made using ArcGIS 10.2. The following acronyms are used for institutions housing the type material in the current study:

**BBSL** USDA ARS Pollinating Insects Research Unit, Logan, Utah.

**CAS** California Academy of Sciences, San Francisco, California. Robert Zuparko.

**SEMC** Snow Entomological Museum Collection, Lawrence, Kansas. Michael Engel and Jennifer Thomas.

All specimens are deposited in the BBSL collection unless otherwise noted.

## Systematics

### Subgenus Procockerellia

Taxon classificationAnimaliaHymenopteraAndrenidae

Timberlake

Perdita (Procockerellia) Timberlake, 1954: 402. Type species: Perdita (Procockerellia) albonotata Timberlake, 1954, by original designation.
Perdita (Allomacrotera) Timberlake, 1960: 131. Type species: Perdita (Procockerellia) stephanomeriae Timberlake, 1954, by original designation and monotypy. **Syn. n.**

#### Subgeneric diagnosis.

**Figure 1.**
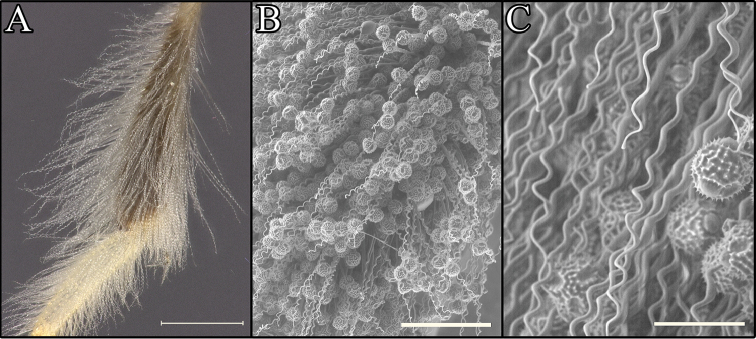
Tibial scopal hairs of *Procockerellia*. **A** *Perdita albonotata* (BBSL972407) **B** *P. moabensis* (36765 (BBSL)) **C** *P. moabensis* (36765 (BBSL)). Scale bars: 500 µm (**A**), 200 µm (**B**), 50 µm (**C**).

**Figure 2.**
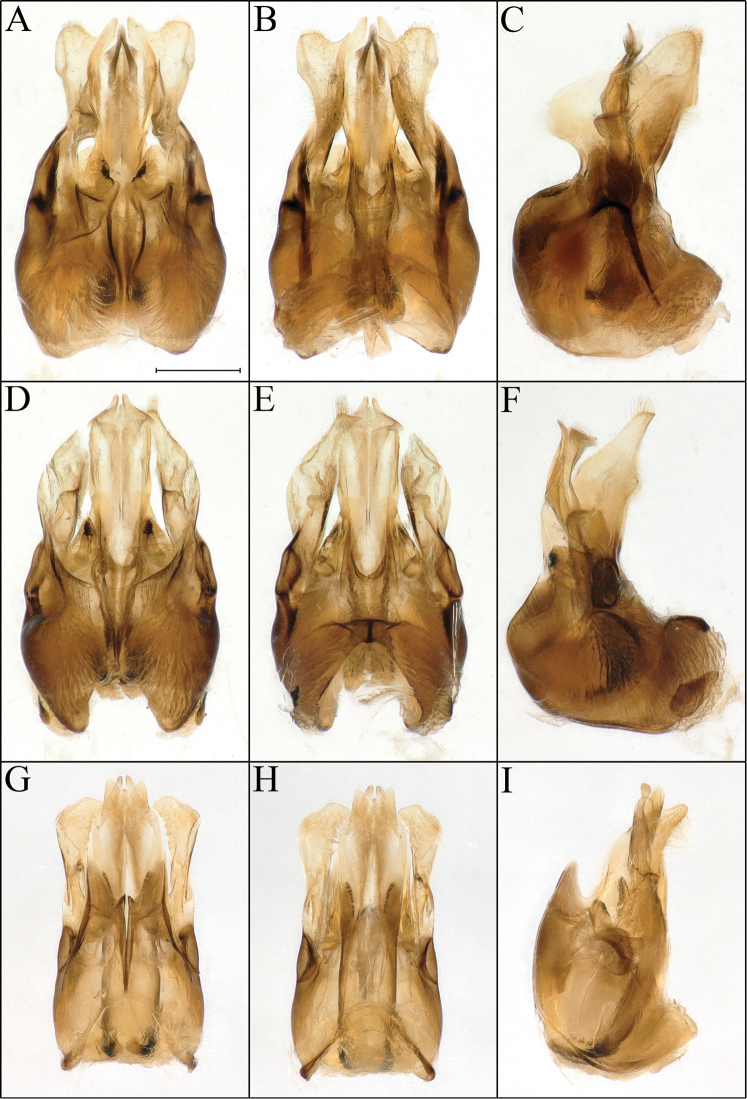
*Procockerellia* male genitalia. *Perdita albonotata* (BBSL529462) **A** dorsal view **B** ventral view **C** lateral view. *Perdita moabensis* (BBSL779598) **D** dorsal view **E** ventral view **F** lateral view. *Perdita stephanomeriae* (BBSL317528) **G** dorsal view **H** ventral view **I** lateral view. Scale bar: 250 µm, all images are the same scale.

*Procockerellia* can be recognized by two characters. First, the unique scopal hairs of the female are long, dense, and tightly corkscrew-shaped, appearing kinky or crimped (Fig. 1). Second, the male S8 is apically narrowed into a carinate median keel (Fig. 2), rather than having a club-shaped apical process as found in related and similar subgenera *Cockerellia* Ashmead, 1898, *Hexaperdita* Timberlake, 1954, and *Pentaperdita* Cockerell and Porter, 1899. *Callomacrotera* Timberlake, 1954 also has a median carina on S8, but the apical process is short and spade-shaped (rather than long and narrow), and the subgenus can be separated by numerous other morphological characters (Timberlake 1954). *Procockerellia* can be further recognized by having the maxillary palpi 3- or 5-jointed in both sexes, mandibles expanded medially in the females and female pygidial plate truncate, lacking a median emargination. The male hind tarsal claws can be either simple or bidentate.

#### Biology.

Although specimens of *Procockerellia* have been collected on many plant families (see below), our results support the idea that all the species are specialists on the plant genus *Stephanomeria* Nutt. (Asteraceae), since only *Stephanomeria* pollen has been found in the scopae of all three species. Bees are active in the early morning before the flowers close, and may also be active in the evening. The nesting biology is unknown, but they are presumably ground nesting bees like other species of *Perdita*. Both *P. albonotata* and *P. moabensis* are found throughout the flowering season from spring to fall, suggesting they are multivoltine. Thus, the flight period of *Procockerellia* matches the bloom period of the genus *Stephanomeria*, which contains species that collectively bloom from spring to fall (Gottlieb 1972). The paucity of collection events renders the phenology of *P. stephanomeriae* unclear.

#### Remarks.

The relationship between *Procockerellia, Allomacrotera* and closely-related subgenera is ambiguous, though *Prockerellia* is clearly a member of the monophyletic group made up of the subgenera *Callomacrotera*, *Cockerellia*, *Hexaperdita*, *Pentaperdita* and *Xeromacrotera* Timberlake, 1954 (Danforth 1996). The reduced number of maxillary palpi suggests an affinity to the subgenus Pentaperdita, which has the maxillary palpi 5-jointed (Timberlake 1954). Danforth (1996) suggested a close relationship to *Cockerellia*, though the species of *Procockerellia* also bear a general resemblance to the monotypic subgenus Xeromacrotera, which also has an uncertain phylogenetic relationship (Portman et al. 2016a).

**Figure 3.**
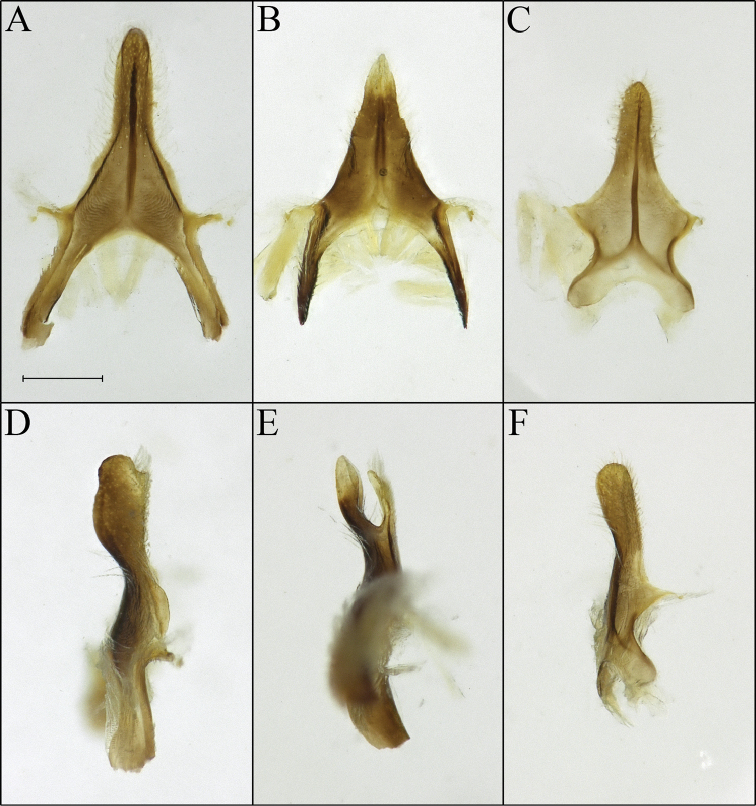
*Procockerellia* male S8. **A** *Perdita albonotata* (BBSL529469) ventral view **B** *P. moabensis* (BBSL779598) ventral view **C** *P. stephanomeriae* (BBSL317528) ventral view **D** *P. albonotata* lateral view **E** *P. moabensis* lateral view **F** *P. stephanomeriae* lateral view. Scale bar: 250 µm, all images are the same scale.

**Figure 4.**
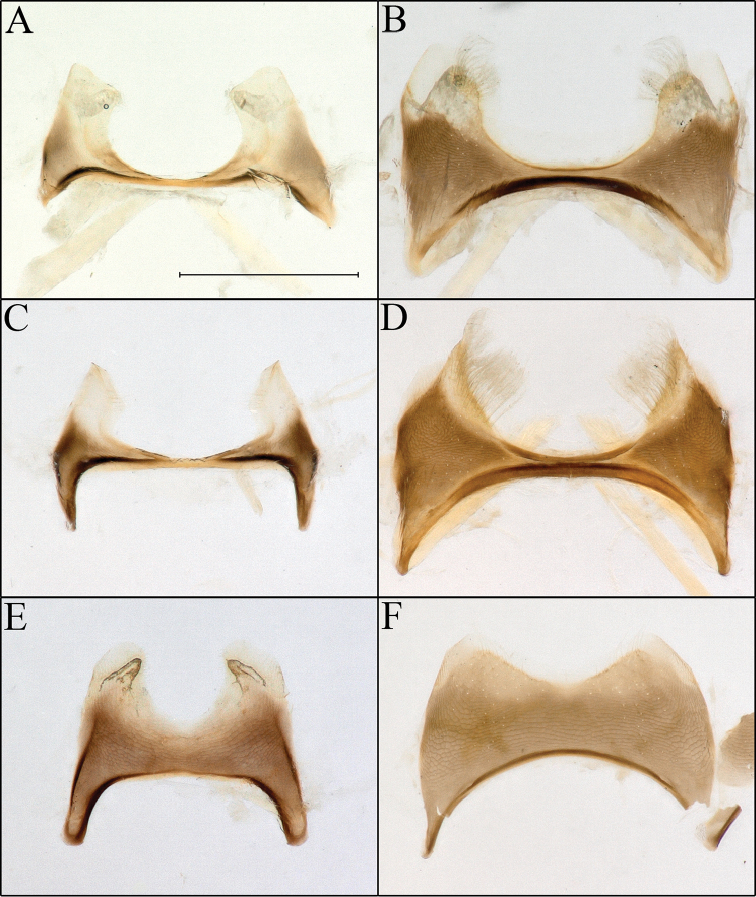
*Procockerellia* male S7 and S6. *Perdita albonotata* (BBSL529462) **A** S7 **B** S6. *Perdita moabensis* (BBSL779598) **C** S7 **D** S6. *Perdita stephanomeriae* (BBSL317528) **E** S7 **F** S6. Scale bar: 500 µm, all images are the same scale.

**Figure 5.**
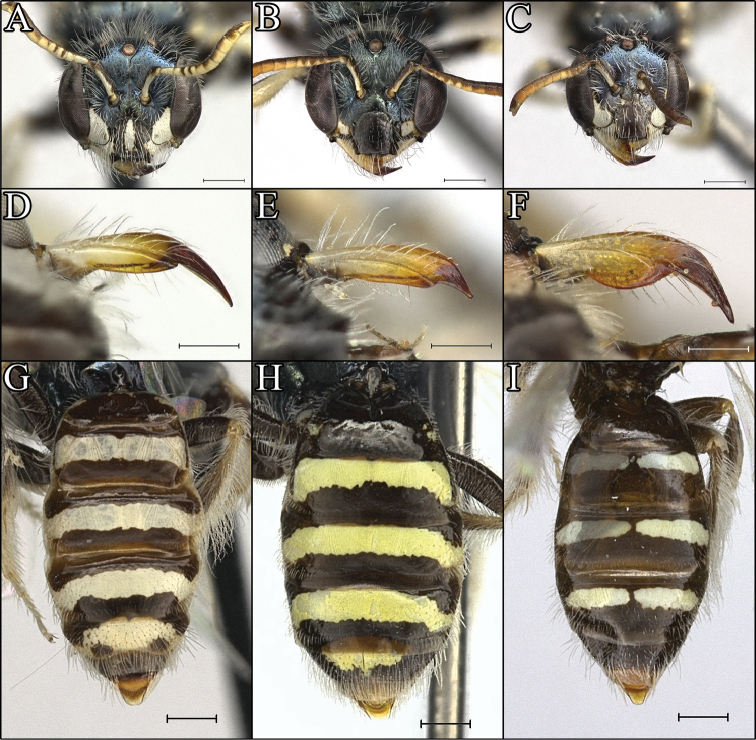
*Procockerellia* females. Faces: **A** *Perdita albonotata* (BBSL668188) **B** *P. moabensis* (BBSL23824) **C** *P. stephanomeriae* (BBSL531898). Scale bars = 500 µm. Mandibles: **D** *P. albonotata* (BBSL640552) **E** *P. moabensis* (BBSL515687) **F** *P. stephanomeriae* (BBSL317523). Scale bars = 250 µm. Metasomas: **G** *P. albonotata* (BBSL311790) **H** *P. moabensis* (BBSL471657) **I** *P. stephanomeriae* (BBSL531898). Scale bars: 500 µm.

**Figure 6.**
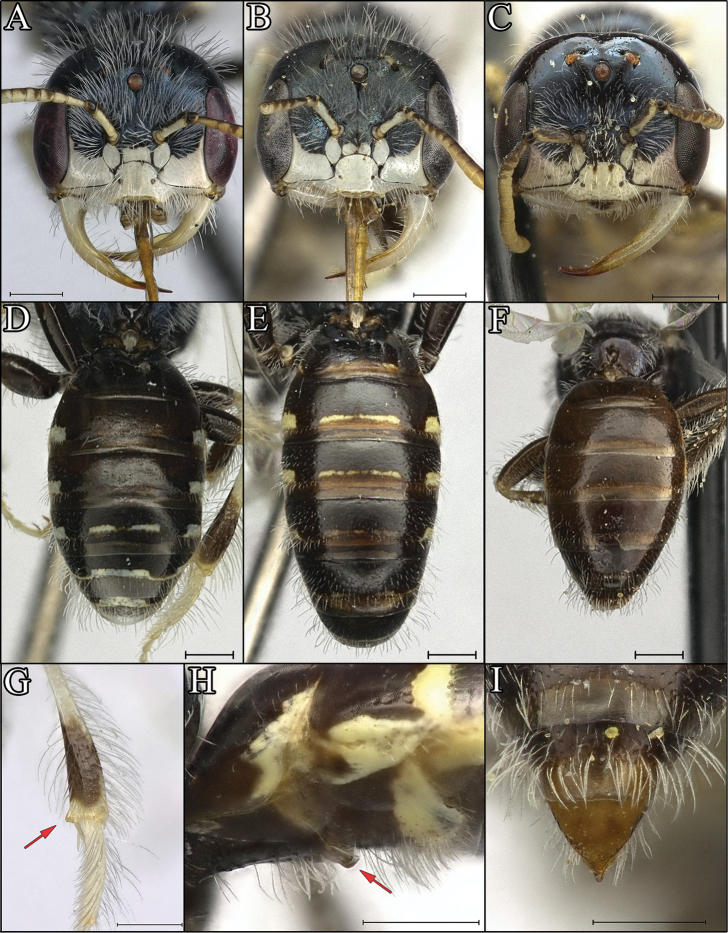
*Procockerellia* males. Faces (of large males): **A** *Perdita albonotata* (BBSL529482) **B** *P. moabensis* (36868 (BBSL)) **C** *P. stephanomeriae* (BBSL317506). Metasomas: **D** *P. albonotata* (BBSL529482) **E** *P. moabensis* (BBSL311790) **F** *P. stephanomeriae* (BBSL317506). Identifying characters: **G** *P. albonotata* tibial nub (BBSL669043) **H** *P. moabensis* S1 flange (BBSL238283) **I** *P. stephanomeriae* pointed pygidial plate (BBSL317506). Scale bars: 500 µm.

The many similarities in scopal hairs (Fig. 1), morphology, genitalia (Fig. 2), apical sterna (Figs 3, 4), coloration and general gestalt (Figs 5, 6) all support a close relationship between *Procockerellia* and *Allomacrotera* that does not justify different subgenera. The structural similarities between S6, S7, and S8 in the males of *P. albonotata* and *P. moabensis* suggest that these are sister species, which would render *Allomacrotera* paraphyletic. In particular, *P. albonotata* and *P. moabensis* have S6 and S7 deeply divided and emarginate and S6 with pronounced lateral hair tufts; these characters are lacking in *P. stephanomeriae* (Figs 3, 4). Timberlake (1980) and Michener (2007) also reported that *Allomacrotera* lacked lateral furrows in the flanks of the pronotum, but all three species contained in the two subgenera have the flanks of the pronotum moderately impressed.

The close relationship between *P. albonotata* and *P. moabensis* suggests two possible solutions to fix the classification of *Procockerellia* and *Allomacrotera*. Either (1) *P. moabensis* should be moved from *Allomacrotera* to *Procockerellia*, or (2) *Allomacrotera* and *Procockerellia* should by merged. If *P. moabensis* were to be moved to *Procockerellia*, it would eliminate the sole defining character of *Allomactera* (bifurcate hind tarsal claws in the male) because both *P. moabensis* and *P. stephanomeriae* share this character, while *P. albonotata* lacks it. In addition, the only remaining character unique to *Allomacrotera* would be the 3-jointed maxillary palpi. However, we agree with Timberlake (1954) that the reduction of maxillary palpi is more important for classification than the specific number of palpi. Indeed, a similar pattern can be seen in the Halictoides group of subgenus Perdita *sensu stricto*, which includes incredibly similar species in which the maxillary palpi collectively range in number from one to five (Timberlake 1958). Therefore, we have chosen to synonymize *Allomacrotera* with *Procockerellia* due to the shared characters of the corkscrew-shaped scopal hairs (Fig. 1) and keel-shaped apical process of S8 (Fig. 3).

The species of *Procockerellia* are distinctive in *Perdita* due to their unique scopal hairs, which are especially long, dense, and tightly corkscrew-shaped, appearing crimped under all but the highest magnification (Fig. 1). This type of scopal hair morphology is rare, and to our knowledge only occurs in one other group, the panurgine genus *Panurgus* Panzer, 1806 (Pasteels et al. 1983). The corkscrew hairs of *Procockerellia* encircle the hind tibia and basitarsus, and the tips are slightly clavate (Fig. 1C). Scopal hairs are also present on the hind femur and trochanter, though these are minutely branched rather than corkscrew-shaped. Similar to the related subgenera *Callomacrotera*, *Cockerellia*, *Hexaperdita*, *Pentaperdita*, *Xeromacrotera* (Danforth 1996), the species of *Procockerellia* initially pack dry pollen into the scopa and then cap it with pollen that has been moistened with nectar (Timberlake 1954, Norden et al. 1992, Portman and Tepedino 2017). Pollen loads on museum specimens indicate that *P. albonotata* and *P. moabensis* cap approximately the last 20% of the pollen load on the anterior face of the hind tibia with moistened pollen. The pollen on the trochanter, femur, basitarsus, and posterior face of the hind tibia are not moistened. The proportion of moist and dry pollen carried by *P. stephanomeriae* is unknown due to a lack of specimens with full pollen loads.

**Figure 7.**
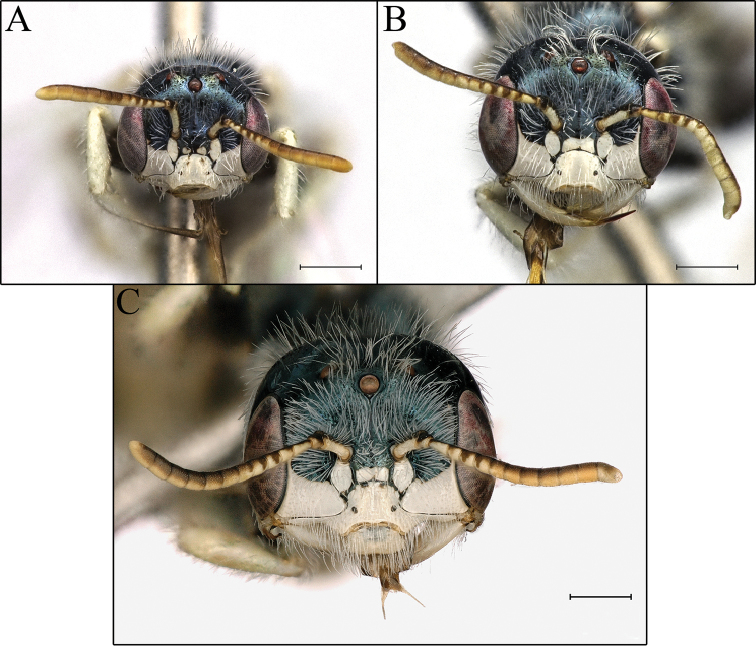
Male head variance in *Perdita albonotata*. **A** small male (BBSL529648) **B** medium male (BBSL532560) **C** large male (BBSL532480). Scale bars: 500 μm, all images are the same scale.

The males of *Procockerellia* vary greatly in size. Similar to many other *Perdita*, the male head size increases and becomes more quadrate with larger body size (fig. 7, Norden et al. 1992, Portman et al. 2016a). However, the distribution of head sizes is continuous, and there are not discrete classes as seen in the Perditini species *Macrotera portalis* (Danforth 1991). The function of the large, quadrate heads is unknown, though it could be used for inter-male aggression and/or grasping females during mating (Norden et al. 1992, Danforth and Neff 1992).

#### Key to species.

Females:

**Table:** 

1	Vertex and frons strongly shining, lacking tessellation (Fig. 5C); metasoma with narrowly-interrupted pale bands extending straight to lateral margins (Fig. 5I); pronotal collar with slight carina dorso-laterally; inner margin of mandible broadly expanded (Fig. 5F); maxillary palpi 3-segmented	***P. stephanomeriae* Timberlake**
–	Vertex and frons (at least laterally and ventrally) with medium or dense tessellation (Fig. 5A–B); pronotal collar with prominent rounded nub apico-laterally; metasoma with pale bands complete and curving apically on lateral margins (Fig. 5G–H); inner margin of mandible not broadly expanded (Fig. 5D–E); maxillary palpi 5-segmented	**2**
2	Frons and vertex heavily tesselate and dullish; face with light marks limited to small, transverse lateral marks, or even absent (Fig. 5B); metasomal bands generally yellowish; pygidial plate broadly truncate apically (Fig. 5H)	***P. moabensis* Timberlake**
–	Frons and vertex slightly tessellate and shining; face with pale, triangular lateral marks reaching level of antennae, clypeus with lateral margins white and often with a median white band (Fig. 5A); pygidial plate slightly narrower and more rounded apically (Fig. 5G); metasomal bands generally white (Fig. 5G), but sometimes yellowish	***P. albonotata*** Timberlake
Males:
1	Frons and vertex strongly tessellate and dull (Fig. 6B); S1 medially with small, outflexed apical margin (Fig. 6H)	***P. moabensis* Timberlake**
–	Frons and vertex weakly tessellate and shining; S1 unmodified	**2**
2	Apex of hind tibia with small nub above tibial spurs (Fig. 6G); T7 broadly rounded, without a point; hind tarsal claws simple; pronotal collar with prominent rounded nub laterally; metasoma generally with white or yellowish markings (Fig. 6D)	***P. albonotata* Timberlake**
–	Apex of hind tibia lacking a nub; T7 ending in a small triangular point (Fig. 6I); hind tarsal claws bidentate; pronotal collar with sharp transverse carina dorso-laterally; metasoma generally lacking markings (Fig. 6F)	***P. stephanomeriae* Timberlake**

### Perdita (Prockerellia) albonotata

Taxon classificationAnimaliaHymenopteraAndrenidae

Timberlake

Figures 1A , 2A–C , 3A, D , 4A–B , 5A, D, G , 6A, D, G , 7 , 8A

Perdita (Procockerellia) albonotata Timberlake, 1954: 403, ♂♀. Holotype female: USA, California, San Bernardino Co., Morongo Valley, 29 September 1944, at flowers of Stephanomeria exigua Nutt. [CAS, type no. 14414]. Examined.

#### Measurements.

Female (n=10): head width 1.5 mm (1.3–1.8 mm), body length 5.8 mm (5.1–6.8 mm). Male (n=11): head width 1.4 mm (1.2–1.7 mm), body length 5.1 mm (4.0–6.2 mm).

#### Diagnosis.

Both sexes of *P. albonotata* have five maxillary palpi and the frons and vertex are weakly tessellate and slightly shining. The female generally has the most extensive facial markings in the subgenus, but the extent is highly variable. In the typical form, the face has the following markings: the clypeus is marked with white on the lateral margins and has a medial white band, the white paraocular marks are broadly triangular and reach the level of the antennal sockets, and the supraclypeal area can be dark or with a pair of white spots (Fig. 5A). The mandible is only slightly expanded on the inner margin and has a long apical tooth (Fig. 5D). The pronotal lobe can be white or dark. The white (sometimes yellowish) abdominal bands on T2–T5 are entire and curve towards the apical margins laterally (Fig. 5G).

The male is unique in *Procockerellia* due to the simple hind tarsal claws and the presence of an apical nub on the anterior face of the hind tibia, just above the tibial spurs (Fig. 6G). In addition, the male has the pygidial plate broadly and evenly rounded, and the discs of terga have small white markings, with the markings generally more pronounced on the apical terga (Fig. 6D).

#### Distribution.

**Figure 8.**
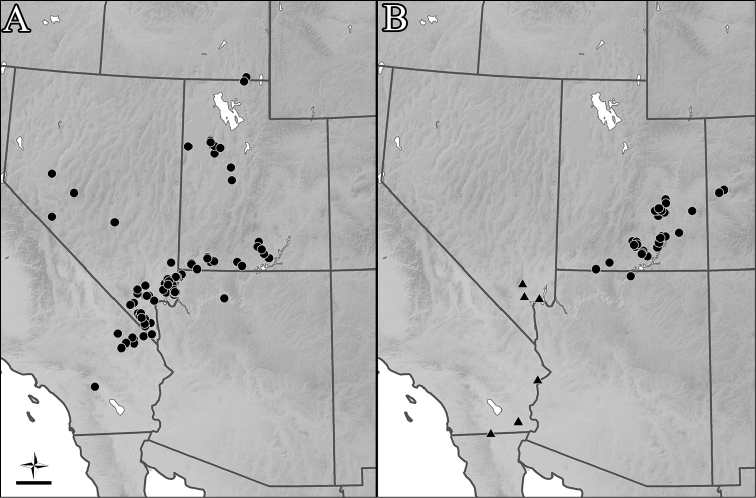
Occurrence maps. **A** *Perdita albonotata* **B** *P. moabensis* (circles) and *P. stephanomeriae* (triangles). Scale bar: 100 km.

Arid regions of the West in Arizona, California, Idaho, Nevada and Utah: Great Basin, southern Colorado Plateau, Mojave Desert (Fig. 8A). There is a single record from the Sonoran Desert. Frequently found in sandy areas.

#### Phenology.

**Table:** 

**Month**:	**Apr**	**May**	**Jun**	**Jul**	**Aug**	**Sep**	**Oct**	**Nov**
# of records	11	2575	529	313	655	243	142	0

#### Floral records.

**Asclepiadaceae** (1 ♂): *Asclepias* sp. 1 ♂, **Asteraceae** (61 ♂ 26 ♀): *Asteraceae* sp. 1 ♂ 1 ♀, *Baileya pleniradiata* 2 ♂, *Grindelia squarrosa* 1 ♂, *Helianthus* sp. 1 ♀, *Isocoma acradenia* 1 ♂, *Malacothrix sonchoides* 3 ♂ 1 ♀, *Pectis papposa* 2 ♂, *Rafinesquia neomexicana* 1 ♂, *Senecio spartioides* 1 ♂, *Stephanomeria exigua* 3 ♂ 3 ♀, *S. pauciflora* 2 ♂ 1 ♀, *S.* sp. 44 ♂ 19 ♀, **Brassicaceae** (6 ♂ 15 ♀): *Streptanthus* sp. 6 ♂ 15 ♀, **Euphorbiaceae** (4 ♂): *Croton* sp. 1 ♂, *C. wigginsii* 3 ♂, **Fabaceae** (1 ♂): *Acacia greggii* 1 ♂, **Loasaceae** (1 ♂): *Mentzelia multiflora* 1 ♂, **Malvaceae** (1 ♂): *Sphaeralcea* sp. 1 ♂, **Polemoniaceae** (1 ♂): *Eriastrum wilcoxii* 1 ♂, **Polygonaceae** (3 ♂): *Eriogonum cernuum* 1 ♂, *E. deflexum* 1 ♂, *E. nummulare* 1 ♂.

#### Additional material examined.

Total specimens: 1403 ♂ 3060 ♀. **USA: ARIZONA: Coconino County**: Colorado River, Fossil Rapids (36.27333 -112.525): 1 ♂, 28 Sep 2001, T.L. Griswold, J. Sterling, *Isocoma acradenia*; **Mohave County**: Littlefield, N (36.88 -113.92): 1 ♂, 8 May 1998, M. Andres, K. Keen, K. Receveur, C. Schultz; Littlefield (36.88 -113.92): 1 ♂, 15 Jun 1983, W.J. Hanson; Mesquite, 8 mi E (36.81 -113.98): 3 ♂, 26 Apr 1973, F.D. Parker, P.F. Torchio. **CALIFORNIA: Riverside County**: Palm Springs (33.832 -116.5453): 1 ♂, 2 May 1953, R.M. Bohart; **San Bernardino County**: (34.868 -115.7731): 2 ♀, 28–29 Apr 2012, collector unknown; 5 ♀, 24–25 May 2012, collector unknown; (34.8794 -115.7808): 3 ♀, 16–17 Aug 2011, collector unknown; 10 ♀, 5–6 Oct 2011, collector unknown; 1 ♀, 23–24 Oct 2011, collector unknown; 1 ♀, 28–29 Apr 2012, collector unknown; 11 ♂ 104 ♀, 24–25 May 2012, collector unknown; Black Canyon (35.1223 -115.394): 2 ♀, 30 May 1998, F.D. Parker; Cedar Canyon (35.16192 -115.44098): 1 ♀, 30 May 1998, F.D. Parker; Hole-in-the-Wall, 2 mi S (35.01783 -115.3843): 7 ♂ 13 ♀, 30 May 1998, F.D. Parker; Kelso, 1 km NE (35.0197 -115.6394): 2 ♂ 3 ♀, 31 May 1998, F.D. Parker. **IDAHO: Franklin County**: Preston (42.09 -111.87): 1 ♂ 1 ♀, 8 Aug 1972, G.E. Bohart. **NEVADA: Churchill County**: Sand Mountain, 25 mi SE Fallon (39.3163 -118.4128): 1 ♀, 13 Jul 1980, L. Hanks; **Clark County**: 0.42 mi E McClanahan Spr. (35.6949 -115.1788): 3 ♂ 1 ♀, 12 May 2004, T.L. Griswold, E. Ahlstrom; 3 ♂ 1 ♀, 25 May 2004, E. Ahlstrom, L. Saul; 1 mi SW Little Virgin Peak (36.5927 -114.21): 3 ♀, 8 Jun 2004, E. Ahlstrom, D. Skandilis; 1.3 mi ESE Mule Spr. (36.0261 -115.5594): 1 ♂, 9 Jun 2004, L. Saul, D. Skandilis; 2.3 mi E Sheep Mtn. (35.7473 -115.2407): 6 ♂ 9 ♀, 12 May 2004, T.L. Griswold, E. Ahlstrom; 3.0 mi SE Rainbow Mtn. (36.0832 -115.4479): 2 ♂, 23 Aug 2004, E. Ahlstrom, *Pectis papposa*; 3.5 mi SW Cow Spr. (35.5382 -115.0665): 14 ♂ 1 ♀, 25 May 2004, S.M. Higbee, D. Skandilis; 3.9 mi SSW Whitney Pocket (36.4669 -114.1537): 3 ♂ 7 ♀, 26 May 2005, R. Andrus, S.M. Higbee; Black Mesa, W (36.1607 -114.789): 1 ♂, 9 Oct 1998, T.L. Griswold, *Eriogonum deflexum*; Black Ridge, 4.6 mi NW (36.5975 -114.3594): 1 ♂ 1 ♀, 21 Apr 2005, R. Andrus, *Stephanomeria pauciflora*; Black Wash (36.4113 -114.0778): 1 ♂, 12 Aug 1998, T.L. Griswold, *Baileya pleniradiata*; Bowman Reservoir, E (36.6222 -114.467): 4 ♂ 7 ♀, 5 Aug 1998, M. Andres, C. Schultz; Christmas Tree Pass, W (35.2708 -114.823): 1 ♂, 6 Jun 1998, F.D. Parker; Corn Creek Springs (36.4407 -115.363): 1 ♀, 28 May 1998, F.D. Parker; Eldorado Valley (35.5697 -114.878): 2 ♂, 10 Jun 1998, M. Andres, K. Keen, K. Receveur, C. Schultz; Fire Canyon Wash (36.4548 -114.5088): 18 ♂ 51 ♀, 5 Aug 1998, M. Andres, C. Schultz; Halfway Wash, 1.8 mi NW Virgin River (36.6859 -114.3322): 1 ♂, 6 May 2004, E. Ahlstrom; Highland Range, NW (35.6703 -115.0715): 1 ♂ 1 ♀, 9 Jun 1998, M. Andres, K. Keen; Hot Creek Valley (36.27417 -115.07056): 2 ♂ 2 ♀, 20 Aug 1998, F.D. Parker; Jean Lake, 2.24 mi ENE (35.814 -115.2051): 1 ♂, 10 May 2005, S.M. Higbee, *Rafinesquia neomexicana*; Jean Lake, NE (35.8067 -115.2233): 1 ♂ 3 ♀, 8 Oct 1998, T.L. Griswold; Jean, N (35.8102 -115.2998): 1 ♂, 8 Oct 1998, T.L. Griswold; Juanita Springs Ranch, S Riverside (36.6383 -114.2478): 1 ♂, 15 May 1983, F.D. & J.H. Parker; Kyle Canyon (36.3268 -115.3418): 1 ♀, 28 May 1998, F.D. Parker; Las Vegas Dunes (36.286 -114.9667): 1 ♂ 28 ♀, 22 May 1998, M. Andres, K. Keen, K. Receveur, C. Schultz; Las Vegas, NE (36.2798 -115.0355): 1 ♂, 7 Oct 1998, T.L. Griswold; Mesquite (36.8055 -114.0664): 1 ♂, 4 Oct 1988, P.F. Torchio, R.W. Rust, *Croton* sp.; 14 ♂ 6 ♀, 8 May 1994, P.F. Torchio, D.F. Veirs, *S.* sp.; Mesquite (36.8144 -114.0703): 3 ♀, 19 Sep 1997, F.D. Parker; Mica Peak (36.3348 -114.1445): 2 ♂ 1 ♀, 7 Jun 1998, T.L. Griswold, F.D. Parker; Mormon Mesa (36.5702 -114.4197): 1 ♂ 1 ♀, 5 Aug 1998, M. Andres, C. Schultz; Mormon Mesa (36.7437 -114.375): 10 ♀, 5 Aug 1998, M. Andres, C. Schultz; Mormon Mesa (36.7447 -114.3778): 36 ♂ 180 ♀, 20 May 1998, M. Andres, K. Keen, K. Receveur, C. Schultz; Mormon Well Rd (36.4355 -115.351): 1 ♀, 15 Sep 1998, W.R. Bowlin; Mormon Well Rd (36.4361 -115.3519): 1 ♂, 15 Sep 1998, W.R. Bowlin; Mormon Well Road (36.534 -115.1067): 6 ♂ 8 ♀, 1 Jul 1998, M. Andres, C. Schultz; Mormon Well Road (36.5492 -115.0995): 6 ♂, 16 Jul 1998, M. Andres, C. Schultz; Mud Wash (36.43 -114.1527): 1 ♀, 19 Sep 1998, W.R. Bowlin; Overton, NE (36.5693 -114.4193): 7 ♂ 5 ♀, 21 May 1998, M. Andres, K. Keen, K. Receveur, C. Schultz; Peek a Boo Canyon (36.5032 -115.1577): 2 ♂ 17 ♀, 16 Jul 1998, M. Andres, C. Schultz; Peek a Boo Canyon (36.5065 -115.1477): 15 ♂ 44 ♀, 16 Jul 1998, M. Andres, C. Schultz; Peek a Boo Canyon (36.515 -115.1327): 8 ♂ 13 ♀, 16 Jul 1998, M. Andres, C. Schultz; Peek a Boo Canyon (36.5325 -115.1128): 12 ♂ 42 ♀, 16 Jul 1998, M. Andres, C. Schultz; Peek-A-Boo Cyn. (36.5522 -115.0991): 1 ♂, 8 Sep 2004, T.L. Griswold, *E. cernuum*; Piute Valley (35.46483 -115.052): 8 ♂ 25 ♀, 10 Jun 1998, M. Andres, K. Keen, K. Receveur, C. Schultz;Piute Valley (35.47266 -115.04816): 4 ♂ 1 ♀, 11 Jun 1998, M. Andres, K. Receveur, *S.* sp.; Pulsipher Wash (36.807 -114.1095): 1 ♂ 2 ♀, 16 Sep 1998, W.R. Bowlin; Pulsipher Wash (36.8073 -114.1143): 4 ♂ 19 ♀, 16 Sep 1998, W.R. Bowlin; Pulsipher Wash (36.8083 -114.1138): 6 ♀, 16 Sep 1998, W.R. Bowlin; Riverside, 4.5 mi SW (36.69137 -114.26056): 1 ♂ 2 ♀, 19 Sep 1997, F.D. Parker; Sandstone Bluffs, E (36.0895 -115.4523): 9 ♂ 24 ♀, 25 Jun 1998, T.L. Griswold; St. Thomas Gap, 0.4 mi E(36.4084 -114.0937): 1 ♂, 20 May 2004, E. Ahlstrom; 109 ♂ 118 ♀, 20 May 2004, S.M. Higbee, E. Ahlstrom; 72 ♂ 100 ♀, 20 May 2004, S.M. Higbee, E. Ahlstrom, D. Skandilis, L. Saul; 1 ♂, 28 Jun 2004, E.D. Rentz, *Eriastrum wilcoxii*; 2 ♀, 27 Apr 2005, S.M. Higbee; 109 ♂ 387 ♀, 12 May 2005, D. Allen, E. Ahlstrom, R. Andrus, S.M. Higbee; 59 ♂ 193 ♀, 25 May 2005, S.M. Higbee, E. Ahlstrom; 1 ♂, 26 May 2005, S.M. Higbee, E. Ahlstrom; 1 ♂, 8 Jun 2005, D. Allen, *S. exigua*; 1 ♂, 8 Jun 2005, S.M. Higbee; 1 ♂, 8 Jun 2005, S.M. Higbee, *Acacia greggii*; 2 ♂, 7 Sep 2005, A. Portoluri, *C. wigginsii*; 1 ♂, 7 Sep 2005, E. North; 1 ♂, 13 Oct 2005, T.L. Griswold, *C. wigginsii*; St. Thomas Gap (36.4023 -114.093): 1 ♂ 4 ♀, 27 May 1998, F.D. Parker; 57 ♂ 59 ♀, 7 Jun 1998, F.D. Parker; 8 ♂ 37 ♀, 12 Aug 1998, M. Andres, T.L. Griswold, C. Schultz; 15 ♂ 46 ♀, 12 Aug 1998, T.L. Griswold, C. Schultz; St. Thomas Gap (36.4041 -114.0933): 1 ♂, 25 May 1998, M. Andres, K. Receveur, C. Schultz, *B. pleniradiata*; 68 ♂ 158 ♀, 26 May 1998, M. Andres, K. Keen, K. Receveur, C. Schultz; 1 ♂, 7 Jun 1998, F.D. Parker; 6 ♂ 15 ♀, 8 Jun 1998, T.L. Griswold, *Streptanthus* sp.; 13 ♂ 54 ♀, 27 Aug 1998, O.J. Messinger, S. Messinger, C. Schultz; 21 ♂ 34 ♀, 6 Oct 1998, T.L. Griswold; 4 ♂ 1 ♀, 6 Oct 1998, T.L. Griswold, *S.* sp.; St. Thomas Gap (36.4058 -114.0937): 19 ♂, 29 Jun 1998, M. Andres, C. Schultz; 4 ♂, 29 Jun 1998, M. Andres, K. Keen, K. Receveur, C. Schultz; St. Thomas Gap (36.4075 -114.0937): 2 ♀, 4 Aug 1998, M. Andres, C. Schultz; 8 ♂ 5 ♀, 18 Sep 1998, W.R. Bowlin; 6 ♂ 30 ♀, 6 Oct 1998, T.L. Griswold; St. Thomas Gap (36.4083 -114.125): 71 ♂ 63 ♀, 11 May–12 Jun 1984, R.C. Bechtel, J.B. Knight; Stewarts Point, NW, 9R (36.3853 -114.4123): 1 ♂, 6 May 1998, M. Andres, K. Keen, K. Receveur, C. Schultz; Tramp Ridge, E (36.3905 -114.1287): 2 ♂ 5 ♀, 4 Aug 1998, M. Andres, C. Schultz; Virgin Mountains, W (36.5768 -114.2042): 1 ♂, 6 Oct 1998, T.L. Griswold; Virgin Valley (36.6868 -114.2643): 1 ♂ 10 ♀, 12 Aug 1998, T.L. Griswold, C. Schultz; 7 ♂ 18 ♀, 6 Oct 1998, T.L. Griswold; Whitney Pocket, NW (36.5448 -114.1765): 1 ♀, 26 May 1998, T.L. Griswold; Whitney Pocket (36.5288 -114.1557): 2 ♂ 7 ♀, 27 May 1998, F.D. Parker; **Lincoln County**: Tule Desert (37.1703 -114.2829): 1 ♂, 17 Aug–30 Sep 1983, R.C. Bechtel, J.B. Knight; 1 ♀, 15 Sep 1983, R.C. Bechtel, J.B. Knight; **Mineral County**: Marrietta, 3 mi S (38.2 -118.3): 1 ♀, 16 Aug 1998, F.D. Parker; **Nye County**: 16 mi E Gabbs (38.8605 -117.623): 26 ♂ 38 ♀, 22 Aug 1998, F.D. Parker; Hot Crk Vly (38.16666 -116.20222): 18 ♂ 19 ♀, 20 Aug 1998, F.D. Parker. **UTAH: Cache County**: Cornish (41.9756 -111.9525): 2 ♂, 4 Aug 1959, G.E. Bohart, *S.* sp.; 1 ♀, 4 Aug 1959, G.E. Bohart, R.A. Nielsen, *S.* sp.; **Garfield County**: Calf Creek (37.7645 -111.4046): 1 ♂, 18 Jun 2003, S.M. Higbee, *S. exigua*; Hole in the Rock Road, Halfway Hollow (37.6338 -111.4449): 7 ♂ 8 ♀, 5–19 Jul 2003, H. Ikerd; 1 ♂, 19–29 Jul 2003, H. Ikerd; Twentyfive Mile Wash (37.5596 -111.3048): 1 ♂ 1 ♀, 18 Sep 2003, A. Johansen, *Asteraceae* sp.; **Kane County**: 2.38 mi SE Stave Spr. (37.2344 -112.8769): 5 ♂ 3 ♀, 14–15 Jun 2007, H. Ikerd, K. Davidson; Dry Fork, N (37.441 -111.2307): 1 ♂, 1 Jul 2003, C. Boyers, *Mentzelia multiflora*; 1 ♂, 18 Sep 2003, J. Tolliver, *S. exigua*; Kitchen Corral Spr., 1.0 mi N (37.2298 -112.1165): 1 ♀, 6 Aug 2003, S.M. Higbee; 1 ♂, 6 Aug 2003, S.M. Higbee, *Senecio spartioides*; Paria River W, on HWY 89 (37.12655 -111.95152): 2 ♀, 21 Aug 2008, T.L. Griswold; Sooner Rocks, 0.6 mi WNW (37.333 -111.0713): 3 ♀, 4 Jun 2003, H. Ikerd; **Millard County**: Oak City (39.37 -112.33): 1 ♀, 24 Jun 1949, G.E. Bohart, *Helianthus* sp.; **Tooele County**: 0.5 mi E Wig Mountain (40.3183 -113.0553): 1 ♀, 6 Jul 2005, E. Jarrell, J.S. Wilson; 1 ♀, 6 Jul 2005, J. Wilson; 1 ♂, 15 Aug 2005, J. Wilson, *Grindelia squarrosa*; 0.6 mi NW Little Granite Mt. (40.2038 -112.845): 1 ♂, 10 Jun 2003, R. Andrus, *S. pauciflora*; 0.7 mi NW Little Granite Mt. (40.2057 -112.8456): 2 ♂ 1 ♀, 10 Jun 2003, O.J. Messinger, *S.* sp.; 5 ♂ 4 ♀, 10 Jun 2003, O.J. Messinger, R. Andrus; 2 ♂ 2 ♀, 20 Jun 2003, O.J. Messinger, C. Boyers; 3 ♀, 18 Jul 2003, R. Andrus, C. Boyers; 1 ♂, 9 Sep 2003, O.J. Messinger, H. Ikerd; 1 ♂ 4 ♀, 4 Aug 2005, J.S. Wilson, L. Wilson; 17 ♂ 60 ♀, 22 Aug 2005, J.S. Wilson; 17 ♂ 26 ♀, 14 Sep 2005, O.J. Messinger, K.T. Huntzinger; 7 ♀, 29 Sep 2005, K.T. Huntzinger, T.L. Griswold; 1.3 mi W Little Granite Mt. (40.1977 -112.8612): 4 ♂ 3 ♀, 10 Jun 2003, O.J. Messinger, R. Andrus; 1 ♀, 10 Jun 2003, R. Andrus, *Malacothrix sonchoides*; 2 ♂ 3 ♀, 20 Jun 2003, O.J. Messinger, C. Boyers; 1 ♀, 1 Jul 2003, J.S. Wilson, O.J. Messinger; 4 ♂, 18 Jul 2003, R. Andrus, C. Boyers; 17 ♂ 33 ♀, 22 Aug 2005, J.S. Wilson; 1 ♂, 14 Sep 2005, K.T. Huntzinger, O.J. Messinger; 10 ♂ 20 ♀, 14 Sep 2005, O.J. Messinger, K.T. Huntzinger; 2 ♂ 5 ♀, 29 Sep 2005, K.T. Huntzinger, T.L. Griswold; 1.8 mi WNW Simpson Butte (40.082 -112.9347): 1 ♂ 6 ♀, 23 Jun 2005, J.S. Wilson, E. Jarrell; 2 mi N Little Granite Mt. (40.225 -112.833): 2 ♂ 3 ♀, 10 Jun 2003, O.J. Messinger, R. Andrus; 1 ♂, 1 Jul 2003, J.S. Wilson, O.J. Messinger; 6 ♂ 24 ♀, 22 Aug 2005, J.S. Wilson; 1 ♂, 14 Sep 2005, O.J. Messinger, K.T. Huntzinger; 2.8 mi NNW Little Granite Mtn. (40.238 -112.8495): 2 ♂, 5 Jul 2005, J.S. Wilson, *S.* sp.; 1 ♂, 5 Jul 2005, J.S. Wilson, E. Jarrell; 2.8 mi W Simpson Buttes (40.0698 -112.9366): 18 ♂ 5 ♀, 17 Jul 2003, R. Andrus, C. Boyers; 26 ♂ 5 ♀, 30 Jul 2003, J.S. Wilson, C.M. Davidson; 3 ♀, 13 Jun 2005, J.S. Wilson, *S. exigua*; 2 ♂ 12 ♀, 13 Jun 2005, J.S. Wilson, E. Jarrell; 4 ♂ 2 ♀, 23 Jun 2005, E. Jarrell,*S.* sp.; 2 ♂, 23 Jun 2005, J.S. Wilson, *M. sonchoides*; 10 ♂ 4 ♀, 23 Jun 2005, J.S. Wilson, *S.* sp.; 15 ♂ 21 ♀, 23 Jun 2005, J.S. Wilson, E. Jarrell; 1 ♂, 8 Jul 2005, E. Jarrell, *S.* sp.; 1 ♂, 8 Jul 2005, J. Wilson, E. Jarrell; 6 ♂, 20 Jul 2005, J.S. Wilson, E. Jarrell; 24 ♂ 21 ♀, 22 Aug 2005, J.S. Wilson; 4 ♂ 3 ♀, 30 Aug 2005, O.J. Messinger; 1 ♂ 2 ♀, 15 Sep 2005, K.T. Huntzinger, *S.* sp.; 1 ♂, 15 Sep 2005, O.J. Messinger, *E. nummulare*; 5 ♂ 21 ♀, 15 Sep 2005, O.J. Messinger, K.T. Huntzinger; 1 ♀, 28 Sep 2005, T.L. Griswold, *S.* sp.; 11 ♂ 33 ♀, 28 Sep 2005, T.L. Griswold, K.T. Huntzinger; 3.5 mi N Wig Mt. (40.3648 -113.088): 5 ♂ 11 ♀, 19 Jun 2003, O.J. Messinger, C. Boyers; 2 ♂ 1 ♀, 17 Jul 2003, R. Andrus, C. Boyers; 4.17 mi SE Wig Mt.(40.2779 -113.0068): 1 ♂, 6 Jul 2005, E. Jarrell; 1 ♂ 1 ♀, 6 Jul 2005, J. Wilson, E. Jarrell; 7 ♂ 3 ♀, 15 Aug 2005, J.S. Wilson, E. Jarrell; 1 ♂, 13 Sep 2005, O.J. Messinger, K.T. Huntzinger; 1 ♂, 26 Sep 2005, T.L. Griswold, K.T. Huntzinger; 4.5 mi SSW White Rock (40.2691 -112.9495): 2 ♀, 1 Aug 2003, J.S. Wilson, C.M. Davidson; 2 ♂, 5 Jul 2005, E. Jarrell, J.S. Wilson; 2 ♂ 1 ♀, 5 Jul 2005, J. Wilson, E. Jarrell; 1 ♂, 5 Jul 2005, J.S. Wilson, *Sphaeralcea* sp.; 2 ♂ 1 ♀, 20 Jul 2005, J.S. Wilson, E. Jarrell; 4.6 mi WSW Little Granite Mtn. (40.1774 -112.9218): 7 ♂ 12 ♀, 23 Jun 2005, J.S. Wilson, E. Jarrell; 1 ♂, 8 Jul 2005, J. Wilson, E. Jarrell; 1 ♂ 3 ♀, 20 Jul 2005, J.S. Wilson, E. Jarrell; 1 ♂ 14 ♀, 22 Aug 2005, J.S. Wilson; 2 ♀, 30 Aug 2005, O.J. Messinger; 2 ♂ 6 ♀, 15 Sep 2005, O.J. Messinger, K.T. Huntzinger; 2 ♂ 2 ♀, 28 Sep 2005, T.L. Griswold, K.T. Huntzinger; 6.3 mi N Wig Mt. (40.4007 -113.0909): 1 ♂, 21 Jun 2005, J.S. Wilson, E. Jarrell; 1 ♂ 1 ♀, 18 Jul 2005, J.S. Wilson, E. Jarrell; 3 ♂, 15 Aug 2005, J.S. Wilson, E. Jarrell; Camels Back Ridge, 3 mi NNE (40.1705 -112.9314): 1 ♂ 1 ♀, 18 Jul 2003, J.S. Wilson, C.M. Davidson; Camels Back Ridge, 3 mi NNE (40.1706 -112.9297): 1 ♂, 12 Jun 2003, R. Andrus, *M. sonchoides*; 13 ♂, 17 Jun 2003, J.S. Wilson; 1 ♂ 5 ♀, 30 Jul 2003, J.S. Wilson, C.M. Davidson; Dugway Proving Grounds, Cedar Ridge, S (site 7) (40.2519 -112.821): 1 ♂, 24 Jun 1997, T. Toler; Dugway Proving Grounds, Dugway, 7 km W (site 6)(40.2333 -112.8313): 4 ♂ 18 ♀, 24 Jun 1997, T. Toler; Dugway Proving Grounds, East Dugway Dunes (40.22111 -112.74361): 1 ♀, 26 Jun 2003, R.L. Johnson; Dugway Proving Grounds, N; Tabbys Peak, 9 km SW (site 13) (40.4296 -113.0946): 10 ♂ 9 ♀, 24 Jun 1997, T. Toler; 3 ♂ 3 ♀, 1 Jul 1997, T. Toler; Dugway Proving Grounds, North Wig Dunes (site 12) (40.22111 -112.74361): 1 ♀, 28 Jul 1997, T. Toler; Dugway Proving Grounds, Wig Mtn., 4.5 km NE (site 8) (40.355 -113.0484): 1 ♂ 2 ♀, 3 Jun 1997, T. Toler; 1 ♂, 24 Jun 1997, T. Toler; Dugway Proving Grounds, Wig Mtn., 8 km E (site 9) (40.2975 -112.9694): 1 ♀, 10 Jun 1997, T. Toler; 1 ♂ 3 ♀, 24 Jun 1997, T. Toler; 2 ♂, 1 Jul 1997, T. Toler; Dugway Proving Grounds, dunes N Wig Mtn. (40.3615 -113.0856): 1 ♀, 18 Jun 2003, R.L. Johnson; 2 ♀, 26 Jun 2003, R.L. Johnson; Dugway Proving Grounds; Dog Area (Ditto), 1 km W (site 19B) (40.1741 -112.9191): 1 ♂ 1 ♀, 3 Jun 1997, T. Toler; Dugway Proving Grounds; Dog Area (Ditto), 8 km N (site 19B) (40.2551 -112.9022): 4 ♂ 10 ♀, 24 Jun 1997, T. Toler; 1 ♂, 1 Jul 1997, T. Toler; Dugway Proving Grounds; Dog Area (Ditto), 8.5 km NE (site 1) (40.2373 -113.8452): 1 ♂ 3 ♀, 24 Jun 1997, T. Toler; Dugway Proving Grounds; Dog Area (Ditto), 9 km N (site 21B) (40.2668 -112.9478): 3 ♂ 3 ♀, 3 Jun 1997, T. Toler; 1 ♀, 24 Jun 1997, T. Toler; Dugway Proving Grounds; Wig Flats, S (site 18) (40.17113 -112.94218): 1 ♀, 3 Jun 1997, T. Toler; Dugway Proving Grounds; Wig Mtn., 10 km WNW (site 12) (40.4 -113.0894): 3 ♂ 6 ♀, 24 Jun 1997, T. Toler; 2 ♂ 1 ♀, 1 Jul 1997, T. Toler; 1 ♂, 24 Jul 1997, T. Toler; **Washington County**: 1.12 mi WSW Stave Spr. (37.2558 -112.9235): 1 ♀, 7 Jul 2006, B. Hays, F. Nicklen; Firepit Kn., 0.72 mi E (37.3494 -113.0912): 1 ♀, 24 Apr 2007, H. Ikerd, K. Davidson; Firepit Kn., 0.73 mi SE (37.3459 -113.0922): 1 ♀, 26 Jun 2007, K. Davidson; Kolob Terrace Road, .63mi NW, Tabernacle Dome (37.305 -113.1016): 1 ♀, 31 May 2007, H. Ikerd; Oak Creek Cyn. (37.2131 -112.9961): 1 ♂, 8 Jun 2006, F. Nicklen, *Asclepias* sp.; Paradise Canyon (37.1442 -113.613): 1 ♀, 19 Jun 1983, D. Beck; 1 ♀, 11 Jul 1983, D. Beck; Spendlove Kn., 0.28 mi NNE (37.3404 -113.1059): 1 ♀, 13 Jun 2006, B. Hays, F. Nicklen; 1 ♀, 22 Jun 2006, B. Hays, F. Nicklen; 1 ♀, 13 Jun 2007, H. Ikerd, K. Davidson; St. George, 5 mi N (37.1738 -113.6186): 1 ♀, 22 Jun 1972, F.D. Parker, D. Vincent; Warner Valley; Warner Valley Rd. (37.02541 -113.43376): 25 ♂ 90 ♀, 14–15 May 2012, K. Williams, E. Sadler, D. Denlinger, A. Kelley; 7 ♂ 11 ♀, 15–16 May 2012, K. Williams, E. Sadler, D. Denlinger, A. Kelley; 61 ♂ 372 ♀, 16–17 May 2012, K. Williams, E. Sadler, D. Denlinger, A. Kelley.

### Perdita (Procockerellia) moabensis

Taxon classificationAnimaliaHymenopteraAndrenidae

Timblerlake

**Figure 9.**
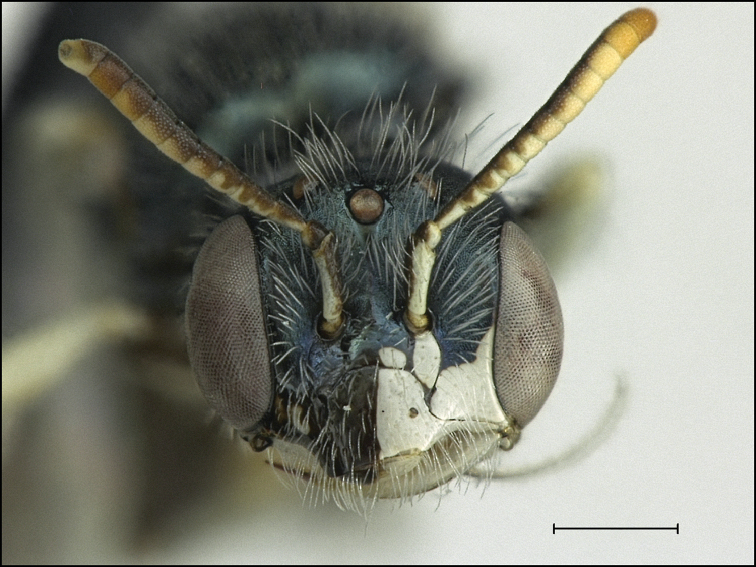
*Perdita moabensis* gynandromorph (BBSL468079). Scale bar: 500 µm.

Figures 1B–C , 2B, E , 4D–E , 5B, E, H , 6B, E, H , 8B , 9

Perdita (Procockerellia) moabensis Timberlake, 1971: 7, ♀; Timberlake, 1980: 15, ♂. Holotype female: USA, Utah, Grand Co., Moab, 8 August 1963, G.F. Knowlton [SEMC]. Examined.
Perdita (Allomacrotera) moabensis ; Timberlake, 1980: 15 (change of subgenus).

#### Measurements.

Female (n=10): head width 1.6 mm (1.5–1.7 mm), body length 6.0 mm (5.3–6.4 mm). Male (n=10): head width 1.7 mm (1.2–1.9 mm), body length 5.8 mm (4.6–6.4 mm).

#### Diagnosis.

Both sexes of *P. moabensis* have the maxillary palpi 5-segmented and the frons and vertex are strongly tessellate and dull. Additionally, the head and mesosoma often have a more greenish (rather than bluish) cast. The female can be recognized by the relatively reduced facial markings which range from transverse lateral marks (Fig. 5B) to entirely absent. In addition, the mandibles are only slightly expanded medially (Fig. 5E) and the metasomal bands are curved towards the apical margins (Fig. 5H).

The male of *P. moabensis* is unique in having the apical margin of S1 slightly flexed out medially (Fig. 6H) as well as S8 bifurcate apically (Fig. 3E). Both characters are subtle but diagnostic in the male, as this is the only *Perdita* species known to have either of these characters. The male can be further distinguished by the strongly tessellate and dull frons and vertex, the evenly rounded pygidial plate, and the bidentate hind tarsal claws (though this may be hard to see).

#### Distribution.

Colorado, Utah and Arizona: Colorado Plateau (Fig. 8B).

#### Phenology.

**Table:** 

Month:	Apr	May	Jun	Jul	Aug	Sep	Oct	Nov
# of records	0	9	16	51	376	70	0	0

#### Floral records.

**Apocynaceae** (2 ♂ 1 ♀): *Cycladenia humilis* 2 ♂ 1 ♀, **Asteraceae** (11 ♂ 19 ♀): *Helianthus annuus* 1 ♂, *Lygodesmia* sp. 1 ♀, *Stephanomeria exigua* 8 ♂ 8 ♀, *S. pauciflora* 2 ♀, *S.* sp. 1 ♂ 3 ♀, *Vanclevea stylosa* 1 ♂ 5 ♀, **Commelinaceae** (1 ♂): *Tradescantia occidentalis* 1 ♂, **Fabaceae** (1 ♂): *Psoralea* sp. 1 ♂, **Lamiaceae** (1 ♂): *Poliomintha incana* 1 ♂, **Malvaceae** (1 ♂): *Sphaeralcea coccinea* 1 ♂, **Polemoniaceae** (3 ♂): *Gilia inconspicua* 2 ♂, *G.* sp. 1 ♂.

#### Additional material examined.

Total specimens: 192 ♂ 327 ♀ 1 gynandromorph. **USA: ARIZONA: Coconino County**: Colorado River, Lee’s Ferry (36.86583 -111.58783): 2 ♀, 9 Jun 2001, L.E. Stevens, *Stephanomeria pauciflora*. **COLORADO: Mesa County**: (39.0295 -108.6276): 3 ♀, 15–16 Jun 2011, collector unknown. **UTAH: Emery County**: Buckskin Springs (38.62 -110.6733): 14 ♂ 26 ♀, 5 Aug 1997, F.D. Parker; Flat Top Pass (38.5417 -110.4906): 1 ♂, 16 Jun 2000, F.D. Parker; Gilson Butte Well (38.5876 -110.583): 27 ♂ 64 ♀, 20 Aug 2001, F.D. Parker; 2 ♀, 22–26 Aug 2001, F.D. Parker, C. Lambkin, M. Metz, M. Hauser; 2 ♂ 2 ♀, 27 Aug 2001, M.E. Irwin, F.D. Parker, M. Metz, M. Hauser, C. Lambkin; Gilson Butte, 4 air mi N (38.64 -110.63): 6 ♂ 22 ♀, 20–23 Jul 1981, D.F. Veirs, T.L. Griswold, F.D. Parker; 1 ♀, 21 Jul 1981, D.F. Veirs, T.L. Griswold, F.D. Parker; 11 ♂ 21 ♀, 5 Aug 1997, F.D. Parker; Gilson Butte, 7 km NW; 30 km N Hanksville (38.6333 -110.6333): 1 ♂ 1 ♀, 21 Aug 2001, F.D. Parker, M.E. Irwin, C. Lambkin, M. Metz, M. Hauser; Goblin Valley turnoff, 25 mi N Hanksville (38.6299 -110.568): 4 ♂ 20 ♀, 16 Sep 1979, F.D. Parker, D.F. Veirs; Goblin Valley, sand dunes (38.64 -110.63): 1 ♂ 3 ♀, 16 Sep 1979, C.L. Hatley, G. Briggs; 12 ♀, 16 Sep 1979, F.D. Parker, D.F. Veirs; Goblin Valley, wash (38.5961 -110.7028): 14 ♀, 16 Sep 1979, F.D. Parker, D.F. Veirs; Iron Wash, 32 km SW Green River (38.7833 -110.4333): 1 ♂, 21 Aug 2001, M.E. Irwin, C. Lambkin, M. Metz, M. Hauser; Little Flat Top, 4 km E (38.5333 -110.45): 2 ♂ 2 ♀, 22 Aug 2001, M.E. Irwin, F.D. Parker, M. Metz, M. Hauser, C. Lambkin; 1 ♂ 9 ♀, 27 Aug 2001, F.D. Parker, C. Lambkin, M. Metz, M. Hauser, M.E. Irwin; 4 ♀, 27 Aug 2001, M.E. Irwin, F.D. Parker, M. Metz, M. Hauser, C. Lambkin; Little Flat Top, 8 km E (38.5167 -110.4333): 1 ♂, 20 Aug 2001, M.E. Irwin, F.D. Parker, M. Metz, M. Hauser, C. Lambkin; 2 ♂ 1 ♀, 22–26 Aug 2001, M.E. Irwin, F.D. Parker, M. Metz, M. Hauser, C. Lambkin; 1 ♂, 27 Aug 2001, M.E. Irwin, F.D. Parker, M. Metz, M. Hauser, C. Lambkin; Little Flat Top (38.5333 -110.4833): 2 ♂ 2 ♀, 20 Aug 2001, F.D. Parker, M.E. Irwin; 1 ♀, 27 Aug 2001, F.D. Parker, C. Lambkin, M. Metz, M. Hauser, M.E. Irwin; 5 ♂ 6 ♀, 27 Aug 2001, F.D. Parker, M.E. Irwin; Little Gilson Butte, 0.5 air mi E (38.5924 -110.594): 9 ♂ 5 ♀, 5 Aug 1997, F.D. Parker; Little Gilson Butte, 0.5 mi E (38.58 -110.59): 1 ♀, 27 Aug 1985, T.L. Griswold, *Vanclevea stylosa*; Little Gilson Butte, 2 air mi E(38.5917 -110.5737): 2 ♂ 6 ♀, 24–26 Aug 1981, D.F. Veirs, T.L. Griswold, F.D. Parker, *S. exigua*; 1 ♂, 25 Aug 1981, D.F. Veirs, T.L. Griswold, F.D. Parker; 2 ♀, 5 Aug 1997, F.D. Parker; Little Gilson Butte, 2 mi E (38.5917 -110.5737): 26 ♂ 24 ♀, 5 Aug 1997, F.D. Parker; Little Gilson Butte, near (38.58 -110.59): 2 ♂, 21 Aug 1980, A.S. Menke, F.D. Parker; Mollys Castle turnoff (38.5492 -110.6184): 1 ♂ 4 ♀, 28 Aug 1985, T.L. Griswold, *V. stylosa*; San Rafael Desert, Butte (38.0 -110.0): 1 ♂, 31 Jul 2000, F.D. Parker; San Rafael Reef, E edge, 2.8 mi S I-70 (38.88694 -110.44416): 1 ♂, 15 Jun 1991, S. Sipes, W.R. Bowlin, *Cycladenia humilis*; 1 ♀, 16 Jun 1991, S. Sipes, W.R. Bowlin, *C. humilis*; 1 ♂, 17 Jun 1991, S. Sipes, W.R. Bowlin, *C. humilis*; South Temple Wash (38.65 -110.66): 1 ♀, 22 Aug 1989, J. Burner, *Lygodesmia* sp.; South Temple Wash (38.65 -110.6667): 5 ♂ 4 ♀, 21 Aug 2001, M.E. Irwin, C. Lambkin, M. Metz, M. Hauser, F.D. Parker; 4 ♂ 1 ♀, 22–26 Aug 2001, F.D. Parker, C. Lambkin, M. Metz, M. Hauser, M.E. Irwin; 1 ♂, 22–26 Aug 2001, M.E. Irwin, C. Lambkin, M. Metz, M. Hauser; Temple Mountain, 3.5 mi SSE San Rafael Desert (38.638 -110.6636): 2 ♂ 19 ♀, 5 Aug 1997, F.D. Parker; Wild Horse Creek, N Goblin Valley(38.5961 -110.7028): 1 ♂ 4 ♀, 21–23 Jul 1981, D.F. Veirs, T.L. Griswold, F.D. Parker; 1 ♀, 13 Sep 1982, F.D. & J.H. Parker; 1 ♀, 13 Sep 1982, F.D. & J.H. Parker, *S.* sp.; 3 ♀ 1 ♀, 13 Sep 1983, F.D. & J.H. Parker; 1 ♀, 13 Sep 1983, F.D. & J.H. Parker, *S.* sp.; 12 ♂ 4 ♀, 5 Aug 1997, F.D. Parker; Wildhorse Creek, 2 mi E (38.5819 -110.7833): 6 ♂, 21 Aug 2001, collector unknown; Wildhorse Creek, 2 mi N (38.618 -110.675): 3 ♂, 21 Aug 2001, F.D. Parker; Wildhorse Creek (38.5852 -110.7008): 4 ♂ 2 ♀ 1 gynandromorph, 21 Aug 2001, F.D. Parker; **Garfield County**: Alvey Wash, 12 km S Escalante (37.6813 -111.4858): 1 ♂, 24 May 2002, M.E. Irwin, F.D. Parker; Calf Creek (37.7645 -111.4046): 1 ♂, 4 Jul 2001, O.J. Messinger, *Sphaeralcea coccinea*; 1 ♂ 1 ♀, 11 Aug 2003, S.M. Higbee, *S. exigua*; 1 ♂, 23 Sep 2003, S.M. Higbee, *S. exigua*; Duffy Mesa, 2 mi NNW (39.10327 -108.45874): 1 ♂, 17 Jun 2003, H. Ikerd, *S. exigua*; 1 ♂ 1 ♀, 15 Sep 2003, A. Johansen; 1 ♂ 1 ♀, 29 Sep 2003, S.M. Higbee, *S.* sp.; Escalante Riv., jct Death Hollow, 1.3 mi W (37.7808 -111.5353): 1 ♂, 5 Sep 2001, B. Morgan; 1 ♂, 21 Aug 2002, C.M. Davidson, *S. exigua*; 1 ♂ 1 ♀, 13 Aug 2003, O.J. Messinger; Hole in the Rock Road, Halfway Hollow (37.6338 -111.4449): 1 ♂ 8 ♀, 5–19 Jul 2003, H. Ikerd; 1 ♂ 4 ♀, 19–29 Jul 2003, H. Ikerd; Hwy 95, jct Hwy 276, 12 mi S (37.9046 -110.452): 1 ♂, 24 May 2000, F.D. Parker; Hwy 95, jct Hwy 276, 9 mi S (37.9107 -110.577): 2 ♂, 24 May 2000, F.D. Parker; 1 ♂, 24 May 2000, F.D. Parker, *Psoralea* sp.; Red Breaks, 2.3 mi NW (37.6924 -111.379): 1 ♂, 25 Jun 2003, H. Ikerd, *Helianthus annuus*; Ticaboo, 4 mi S (37.6142 -110.7168): 1 ♂, 25 May 2000, F.D. Parker; Ticaboo, 5 mi N (37.7368 -110.6635): 1 ♂, 25 May 2000, F.D. Parker; Woodruff Springs, 54 km S Hanksville (37.8661 -110.6178): 2 ♀, 22–27 May 2002, M.E. Irwin, F.D. Parker; **Kane County**: Billy Pasture, 0.4 mi N (37.2059 -112.2912): 1 ♂, 5 Sep 2003, T.L. Griswold, *Tradescantia occidentalis*; Coral Pink Sand Dunes State Park, all Swales (37.03521 -112.73325): 1 ♂ 1 ♀, 30 Jul 1994, C.B. Knisley, J.M. Hill; Dry Fork, N (37.441 -111.2307): 1 ♂, 18 Sep 2003, J. Tolliver, *S. exigua*; Dry Fork (37.4817 -111.2205): 1 ♂, 5 Jun 2000, J. Grixti, *Poliomintha incana*; 1 ♀, 18 Sep 2003, J. Tolliver, *S. exigua*; Sunset Natural Arch, 0.72 mi NE (37.3822 -111.0374): 1 ♂, 5 Jun 2001, L. Topham, *Gilia inconspicua*; 1 ♂, 5 Jun 2001, R. Andrus, *G. inconspicua*; Twentyfive Mile Wash, 1.5 mi S (37.5322 -111.1788): 1 ♂, 4 Jun 2001, S. Messinger, *S. exigua*; Twentyfive Mile Wash, 1.6 mi S (37.5308 -111.1773): 1 ♂, 4 Jun 2001, S. Messinger, *G.* sp.; **Wayne County**: The Notch, 6 mi N Hanksville (38.45107 -110.68065): 1 ♂, 16 Aug 1992, T.L. Griswold.

#### Gynandromorph.

A single gynandromorph of *P. moabensis* was discovered during the course of this study. It was collected in a pan trap on the 21^st^ of August 2001 at Wildhorse Creek in Emery County, UT by Frank Parker (accession number BBSL468079). The gynandromorph is almost entirely female, except the left side of the head (Fig. 9), the left foreleg, and the left midleg are male. The face is split cleanly down the middle; the female half has an antenna with 12 antennal segments, whereas the male half has 13 antennal segments. The pronotal collar on the male side is slightly more protuberant than the female side, but not as much as in a typical male. The rest of the thorax, including the wings and hind legs, are entirely female. This unique specimen is the first known gynandromorph in *Perdita*. Further, this is the first recorded gynandromorph in Panurginae; all previously known gynandromorphs in the family Andrenidae have been restricted to the genus *Andrena* (Wcislo et al. 2004, Hinojosa-Díaz et al. 2012), although an intersex (blended male and female characters) *Acamptopoeum submetallicum* (Spinola) has been previously documented (Ramos and Ruz 2013).

#### Remarks.

Timberlake (1980) reported that the male of this species had only four maxillary palpi. Examination of many specimens (including the female holotype) reveals that the species always has five maxillary palpi.

### Perdita (Procockerellia) stephanomeriae

Taxon classificationAnimaliaHymenopteraAndrenidae

Timberlake

Figures 2G–I , 3C, F , 4E–F , 5C, F, I , 6C, F, I , 8B

Perdita (Procockerellia) stephanomeriae Timberlake, 1954: 404, ♀; Timberlake 1960: 132, ♂. Holotype female: USA, California, San Diego Co., 12 miles south of Ocotillo, 12 November 1939, P.H. Timberlake, at flowers of Stephanomeria pauciflora [CAS type no. 14720]. Examined.
Perdita (Allomacrotera) stephanomeriae ; Timberlake 1960: 131 (change of subgenus).
Perdita (Hexaperdita) glamis Timberlake, 1980: 16, ♂. Holotype male: USA: California: Imperial Co., Glamis, 13 June 1965, G.E. Wallace [CAS type no. 14544]. Examined. **Syn. n.**

#### Measurements.

Female (n=10): head width 1.5 mm (1.4–1.6 mm), body length 5.6 mm (5.2–6.3 mm). Male (n=4): head width 1.5 mm (1.4–1.6 mm), body length 4.9 mm (4.6–5.2 mm).

#### Diagnosis.

Both sexes have the maxillary palpi 3-jointed (whereas the other two species of *Prockerellia* have 5-jointed maxillary palpi) and the frons and vertex are barely tessellate and strongly shining (e.g. Fig. 6C). The transverse dorso-lateral carina on the pronotal collar found in both sexes is distinctive; other *Procockerellia* have a rounded nub laterally. The female has the face marked with white laterally on the clypeus and a triangular mark on the lateral area (Fig. 5C), similar to lighter females of *P. albonotata*. The female can be further recognized by the broad median expansion of the mandibles (Fig. 5F) and narrowly interrupted metasomal bands that don’t curve to the apical margin laterally (Fig. 5I). The male is unique in having a small point apically on the pygidial plate (Fig. 6I). It can be further distinguished by the bidentate tarsal claws and the lack of light bands on the metasoma (Fig. 6F).

#### Distribution.

Nevada and California: Mojave and Sonoran Deserts (Fig. 8B).

#### Phenology.

**Table:** 

Month:	Apr	May	Jun	Jul	Aug	Sep	Oct	Nov
# of records	0	0	2	0	0	0	15	1

#### Floral records.

**Asteraceae**: *Stephanomeria* sp. 1 ♂ 6 ♀.

#### Additional material examined.

Total specimens: 4 ♂ 12 ♀. **USA: CALIFORNIA: San Bernardino County**: Vidal, 1 mi S (34.1062 -114.50738): 1 ♂ 6 ♀, 6 Oct 1988, T.L. Griswold, *Stephanomeria* sp. **NEVADA: Clark County**: 2.2 mi SSW Mormon Well (36.6165 -115.1111): 1 ♀, 14 Jun 2004, E. Ahlstrom, D. Skandilis; Las Vegas, NE (36.2798 -115.0355): 1 ♂, 7 Oct 1998, T.L. Griswold; Pinto Ridge (36.2422 -114.5493): 2 ♂ 5 ♀, 9 Oct 1998, T.L. Griswold.

#### Remarks.

As a result of this study, *P. stephanomeriae* is hereby returned to its original subgeneric assignment, *Procockerellia*. This species appears rare, especially compared to *P. albonotata* and *P. moabensis*, which can be common and locally abundant. Extensive all season sampling conducted in 1998, 2004, 2005 in Clark County, Nevada in the eastern Mojave Desert yielded large numbers of *Procockerellia*. It is therefore interesting that while *P. albonotata* was widely distributed and abundant, *P. stephanomeriae* was rarely detected.

The holotype of *P. glamis* was examined and found to clearly match *P. stephanomeriae*. The mouthparts of the holotype of *P. glamis* are not exposed, which likely led Timberlake (1980) to incorrectly place and describe the species in subgenus Hexaperdita since he could not see the reduced number of palpi.

### Subgenus Cockerellia Ashmead

*Cockerellia* Ashmead, 1898: 284. Type species: *Perdita hyalina* Cresson, 1878, by original designation.

*Philoxanthus* Ashmead, 1898: 285. Type species: *Perdita beata* Cockerell, 1895, by original designation.

### Perdita (Cockerellia) imbellis

Taxon classificationAnimaliaHymenopteraAndrenidae

Timberlake

Perdita (Cockerellia) imbellis Timberlake, 1968: 21, ♂. Holotype male: USA, Arizona, Coconino Co., 28 May 1954, F. Werner [CAS type no. 13525]. Examined.
Perdita (Cockerellia) hilaris Timberlake, 1968: 2, ♂ (syn. Timberlake 1980). Holotype male: USA, Utah, Dixie State Park, 13 June 1961, G.E. Bohart [CAS type no. 14554]. Not examined.
Perdita (Procockerellia) brachyglossa Timberlake, 1971: 6, ♀. Holotype female: USA, Arizona, Coconino Co., Four and one-half miles southwest of Marble Canyon, 30 August 1967, P.H. Timberlake, on Thelesperma [CAS type no. 14448]. Examined. **Syn. n.**

#### Remarks.

Perdita (Procockerellia) brachyglossa was described from a single female specimen. Timberlake (1971) reported that the female had four maxillary palpi. Examination of the holotype revealed four maxillary palpi on one side and one palp on the other. In every morphological character except the palpi, the holotype of *P. brachyglossa* clearly matches *P. imbellis*. It seems likely that the mouthparts of the holotype of *P. brachyglossa* are either damaged or aberrant.

Due to a *lapsus calami* in Timberlake (1980), *Perdita albomaculata* Timberlake, 1980, *Perdita imbellis* Timberlake, 1968, and *Perdita luculenta* Timberlake, 1968 were all referred to as being in subgenus Cockerellula Strand, 1932. They all belong in subgenus Cockerellia.

### Perdita (Cockerellia) moldenkei

Taxon classificationAnimaliaHymenopteraAndrenidae

Timberlake, 1980

Perdita (Procockerellia) moldenkei Timberlake, 1980: 14, ♂. Holotype male: USA, California, San Diego Co., Ocotillo-Borrego area, 27 March 1972, J.L. Neff, at nectaries of Encelia farinosa [CAS type no. 14614]. Examined.

#### Remarks.

Timberlake’s description of *P. moldenkei* reports the lone type specimen as having five maxillary palpi. However, examination of the type reveals that the specimen clearly has six maxillary palpi on both sides. Timberlake must have miscounted the number of maxillary palpi and described *P. moldenkei* in the incorrect subgenus as a result.

*Perdita moldenkei* is clearly a member of subgenus Cockerellia, and is hereby moved to that subgenus. Based on the examination of the type of *P. moldenkei*, we believe that it is a likely synonym of *P. verbesinae* Cockerell, 1896. However, we have not examined the various syntypes and varieties of *P. verbesinae* in order to confirm this; examination of the syntypes of *P. verbesinae* should be addressed in a broader revision of *Cockerellia*.

## Supplementary Material

XML Treatment for Procockerellia

XML Treatment for Perdita (Prockerellia) albonotata

XML Treatment for Perdita (Procockerellia) moabensis

XML Treatment for Perdita (Procockerellia) stephanomeriae

XML Treatment for Perdita (Cockerellia) imbellis

XML Treatment for Perdita (Cockerellia) moldenkei

## References

[B1] Ashmead WH (1898) Some new genera of bees. Pysche 8: 282–285. https://doi.org/10.1155/1898/70320

[B2] Cockerell TDA (1895) V. New species of bees. Pysche 7(1): 10–12. https://doi.org/10.1155/1895/45305

[B3] Cockerell TDA (1896) The bees of the genus *Perdita* F. Smith. Proceedings of the Academy of Natural Sciences of Philadelphia 48: 25–107.

[B4] Cockerell TDA Porter W (1899) Contributions from the New Mexico Biological Station—VII. Observations on bees, with descriptions of new genera and species. Annals and Magazine of Natural History 7: 403–421. https://doi.org/10.1080/00222939908678225

[B5] Cresson ET (1878) Descriptions of new North American Hymenoptera in the collection of the American Entomological Society. Transactions of the American Entomological Society 7: 61–136.

[B6] Danforth BN (1991) The morphology and behavior of dimorphic males in *Perdita portalis* (Hymenoptera: Andrenidae). Behavioral Ecology and Sociobiology 29: 235–247. https://doi.org/10.1007/BF00163980

[B7] Danforth BN (1996) Phylogenetic analysis and taxonomic revision of the *Perdita* subgenera *Macrotera*, *Macroteropsis*, *Macroterella*, and *Cockerellula* (Hymenoptera: Andrenidae). University of Kansas Science Bulletin 55: 635–692.

[B8] Danforth BN Neff JL (1992) Male polymorphism and polyethism in *Perdita texana* (Hymenoptera: Andrenidae). Annals of the Entomological Society of America 85: 616–626. https://doi.org/10.1093/aesa/85.5.616

[B9] Gottlieb LD (1972) A proposal for classification of the annual species of *Stephanomeria* (Compositae). Madroño 21: 463–481.

[B10] Hinojosa-Díaz IA Gonzalez VH Ayala R Mérida J Sagot P Engel MS (2012) New orchid and leaf-cutter bee gynandromorphs, with an updated review (Hymenoptera, Apoidea). Zoosystematics and Evolution 88: 205–214. https://doi.org/10.1002/zoos.201200017

[B11] Michener CD (2007) The bees of the world. 2nd ed. Johns Hopkins University Press, Baltimore, 953 pp.

[B12] Norden BB Krombein K V Danforth BN (1992) Taxonomic and bionomic observations on a Floridian panurgine bee, Perdita (Hexaperdita) graenicheri Timberlake (Hymenoptera: Andrenidae). Journal of Hymenoptera Research 1: 107–118.

[B13] Panzer GWF (1806) Kritische Revision der Insektenfauna Deutschlands [vol. 2]. Nürnberg, Felssecker, 271 pp.

[B14] Pasteels JM Pasteels JJ De Vos L (1983) Ètude au microscope èlectronique à balayage des scopas collectrices de pollen chez les Panurginae (Hymenoptera, Apoidea, Andrenidae). Archives de Biologie 94: 53–73.

[B15] Portman ZM Griswold T Pitts JP (2016a) Association of the female of Perdita (Xeromacrotera) cephalotes (Cresson), and a replacement name for *Perdita bohartorum* Parker (Hymenoptera: Andrenidae). Zootaxa 4097: 567–574. https://doi.org/10.11646/zootaxa.4097.4.8 27394567 10.11646/zootaxa.4097.4.8

[B16] Portman ZM Neff JL Griswold T (2016b) Taxonomic revision of Perdita subgenus Heteroperdita Timberlake (Hymenoptera: Andrenidae), with descriptions of two ant-like males. Zootaxa 4214: 1–97. https://doi.org/10.11646/zootaxa.4214.1.1 10.11646/zootaxa.4214.1.1 28006789

[B17] Portman ZM Tepedino VJ (2017) Convergent evolution of pollen transport mode in two distantly related bee genera (Hymenoptera: Andrenidae and Melittidae). Apidologie 48: 461–472. https://doi.org/10.1007/s13592-016-0489-8

[B18] Ramos KS Ruz L (2013) First record of intersexual phenotype in Calliopsini bees (Hymenoptera, Apidae, Andreninae): An unusual specimen of *Acamptopoeum submetallicum* (Spinola). Zootaxa 3609: 239–242. https://doi.org/10.11646/zootaxa.3609.2.10 24699586 10.11646/zootaxa.3609.2.10

[B19] Smith F (1853) Catalogue of Hymenopterous Insects in the Collection of the British Museum. Part 1. Andrenidae and Apidae. British Museum, London, 465 pp.

[B20] Strand E (1932) Miscellanea nomenclatorica zoologica et palaeontologica. Folia Zoologica et Hydrobiologica 4: 193–196.

[B21] Timberlake PH (1954) A revisional study of the bees of the genus *Perdita* F. Smith, with special reference to the fauna of the Pacific Coast (Hymenoptera, Andrenidae). Part I. University of California Publications in Entomology 9: 345–432.

[B22] Timberlake PH (1958) A revisional study of the bees of the genus *Perdita* F. Smith, with special reference to the fauna of the Pacific Coast (Hymenoptera, Andrenidae). Part III. University of California Publications in Entomology 14: 303–410.

[B23] Timberlake PH (1960) A revisional study of the bees of the genus *Perdita* F. Smith, with special reference to the fauna of the Pacific Coast (Hymenoptera, Andrenidae). Part V. University of California Publications in Entomology 17: 1–156.

[B24] Timberlake PH (1968) A revisional study of the bees of the genus *Perdita* F. Smith, with special reference to the fauna of the Pacific Coast (Hymenoptera, Andrenidae). Part VII. University of California Publications in Entomology 49: 1–196.

[B25] Timberlake PH (1971) Supplementary studies on the systematics of the genus *Perdita* (Hymenoptera: Andrenidae). University of California Publications in Entomology 66: 1–63.

[B26] Timberlake PH (1980) Supplementary studies on the systematics of the genus *Perdita* (Hymenoptera, Andrenidae). Part II. University of California Publications in Entomology 85: 1–65.

[B27] Wcislo WT Gonzalez VH Arneson L (2004) A review of deviant phenotypes in bees in relation to brood parasitism, and a gynandromorph of *Megalopta genalis* (Hymenoptera: Halictidae). Journal of Natural History 38: 1443–1457. https://doi.org/10.1080/0022293031000155322

